# Laparoscopic Radical Resection of Colorectal Cancer in the Treatment of Elderly Colorectal Cancer and Its Effect on Gastrointestinal Function

**DOI:** 10.3389/fsurg.2022.840461

**Published:** 2022-02-24

**Authors:** Biao Liu, Chuanhui Yao, Haiying Li

**Affiliations:** ^1^The Third Department of Surgery, Cangxian Hospital, Cangzhou, China; ^2^The First Department of Surgery, Cangxian Hospital, Cangzhou, China; ^3^Department of Medical Affairs, Cangxian Hospital, Cangzhou, China

**Keywords:** colorectal cancer, laparoscopic, efficacy, safety, gastrointestinal tract function

## Abstract

**Objective:**

To explore the efficacy and safety of laparoscopic radical resection of colorectal cancer in the elderly patients and its impact on gastrointestinal function.

**Methods:**

A total of 122 elderly patients with colorectal cancer admitted to our hospital from March 2020 to June 2021 were selected as the research subjects, and they were divided into the control group (*n* = 61) and the observation group (*n* = 61). The control group was treated with traditional laparotomy, and the observation group was treated with laparoscopic radical resection of colorectal cancer. The clinical data of operation time, incision length, intraoperative bleeding volume, and hospitalization time in the two groups were recorded. Serum motilin (MTL) and gastrin (GAS) levels were measured pre- and post-operatively. The duration of abdominal distension, the time for the abdominal sound to return to normal, the time for the anal exhaust to normal, and the time for normal food intake were recorded after operation. The patients were followed up for 6 months post-operatively, and the complications during follow-up were recorded.

**Results:**

The total response rate of the observation group (95.08%) was higher than that of the control group (81.97%) (*P* < 0.05). The operation time, incision length, intraoperative bleeding volume, and hospitalization time of the observation group were lower than those of the control group (*P* < 0.05). The duration of abdominal distension, the time for bowel sounds to return to normal, the time for the anus to exhaust gas to normal, and the normal eating time in the observation group were all lower than those in the control group (*P* < 0.05). After surgery, the levels of MTL and GAS in the two groups were lower than those before surgery, and those in the observation group were lower than those in the control group (*P* < 0.05). The total incidence of complications in the observation group (3.28%) was lower than that in the control group (13.12%) (*P* < 0.05).

**Conclusion:**

Laparoscopic radical resection of colorectal cancer in the elderly patients has good effect, short operation time, less trauma, less blood loss during operation, short hospital stay, good recovery of gastrointestinal function, fewer complications, and high safety.

## Introduction

The mortality rate of colorectal cancer ranks in the forefront of malignant tumors. The majority of patients with colorectal cancer are elderly patients, with more senile diseases and high post-operative complications (1–3). Surgical radical surgery is the preferred treatment for colorectal cancer (4, 5). Open surgery for colorectal cancer has a definite effect, but this type of surgery has the following disadvantages: (1) patients with large opening have a large amount of bleeding during the operation, larger hidden fluid loss, unstable internal environment, and obvious stress reaction; (2) post-operative patients will have varying degrees of intestinal inflammation, which is a potential adverse effect on the patients' post-operative rehabilitation and nursing care; (3) studies have shown that patients undergoing traditional colorectal surgery suffer from prolonged post-operative pain, prolonged hospitalization, long recovery time for severe intestinal inflammation, long exhaust time, and prolonged initial ambulation (6–8). However, due to the large incision, clear intraoperative vision, and clear anatomical position, open surgery can often get satisfactory surgical results (9, 10). In recent years, with the clinical application and development of the concept of “minimally invasive,” laparoscopic surgery has been widely used. With the improvement of surgical methods and the maturity of surgical skills, laparoscopic radical surgery has been able to achieve similar short-term curative effect as open surgery (11, 12). The purpose of this study was to investigate the efficacy, safety, and gastrointestinal effects of laparoscopic radical resection for colorectal cancer in the elderly patients.

## Materials and Methods

### Patients

A total of 122 elderly patients with colorectal cancer who were admitted to our hospital from March 2020 to June 2021 were selected as the research subjects. There were 74 males and 48 females, with the average age of (69.08 ± 4.36) years old. Inclusion criteria were as follows: Age > 60 years old; the patient's symptoms and pathological biopsy diagnosis were consistent with the diagnostic criteria for colorectal cancer (13); patients who have not received chemoradiotherapy or immunotherapy before operation. Exclusion criteria were as follows: Patients with a previous history of conversion from laparoscopic to laparotomy; patients with coagulation disorder; patients with hematological diseases; severe heart, liver, lung, and renal insufficiency; patients with gastrointestinal bleeding and intestinal obstruction; patients who have a history of chemotherapy or use drugs that affect gastrointestinal motility and hormones; patients who fell off during follow-up. All the patients were divided into the control group (*n* = 61) and the observation group (*n* = 61) according to the random number table. There was no significant difference in general information such as gender and age between the two groups (*P* > 0.05), as shown in Table 1.

**Table 1:** Comparison of general information between two groups.

**Group**	**Gender**		**Age (years)**	**TNM staging**			**Tumor diameter (cm)**	**Tumor location**	
	**Male**	**Female**		**I**	**II**	**III**		**Colon cancer**	**Rectal cancer**
Control group	39	22	68.89 ± 4.18	26	23	12	3.49 ± 0.51	36	25
Observation group	35	26	69.30 ± 4.98	22	25	14	3.54 ± 0.53	38	23
t/χ^2^	0.549	0.493	0.571	0.531	0.137
*P*	0.459	0.623	0.752	0.596	0.711

### Treatment Methods

Before surgery, both the groups were given correct anemia, given to maintain water and electrolyte, acid-base balance, and strengthen nutritional supplements. Two days before surgery, half-flow diet was adopted, 1 day before surgery, full-flow diet was adopted, oral intestinal antibiotics were taken from 3 days before surgery, fasting for 12 h before surgery, no drinking for 8 h before surgery, indwelling catheterization was given before surgery, and general enema was given in the morning before surgery to empty intestinal contents.

The control group was treated with traditional laparotomy: All patients were treated with endotracheal intubation and intravenous combined general anesthesia. The position was supine or lithotomy, and the specific incision location was selected according to the tumor site, such as the middle incision of the lower abdomen for sigmoid colon surgery. For the operation of the right hemicolon and descending colon, the midabdominal incision around the umbilicus or the incision through the rectus abdominis was selected. An incision of about 10 cm was made in the abdomen of the patient, through which the mesentery was cut, and the corresponding intestinal segment of colon cancer was removed and ligation was performed with surgical instruments such as forceps. Meanwhile, the lymph nodes in the region were cleared, and the intestinal tube was cut off at 5 cm from the lesion to complete anastomosis.

The observation group was treated with laparoscopic radical resection of colorectal cancer: First, intubation was inserted into the trachea of the patient after general anesthesia, the patient was supine on the sterilized bed sheet, and the parameters of pneumoperitoneum were set with the pressure of 13 mmHg, and an observation hole was set at 10 mm below the umbilicus and 3 mm at the left and right lateral abdominal edge of the umbilicus. An operation hole was set at the left and right McBurney's points with a size of 10 ~ 11 mm, and then the laparoscope was tilted 30° for observation to determine whether there was organ metastasis in the abdominal cavity and whether the tumor eroded the serosal membrane. A cotton tape was placed in the intestine at 9 mm near the tumor, and it was suspended and stretched. Afterward, an ultrasonic knife is used to cut the junction of the peritoneum and the sigmoid mesocolon. During the cutting process, the gap between the loose connective tissue at the root of the membrane and the sigmoid mesocolon can be separated, thereby effectively protecting the ureter. Then, the lymph nodes and the blood vessels under the mesentery were anatomized, so that the sub mesenteric membrane was exposed, and the root of the vein was broken and tied using Hemolock. The thin membrane of blood vessel and intestine under direct vision was disconnected to ensure that the pelvic fascia wall layer is not damaged. The lymph nodes, fat, and connective tissue were cleaned and the anterior fascia behind the rectum was separated until it reaches the levator ani muscle. After segmentation of the anal tail facing band, the sacral fascia, and the coccyx muscle, the mesorectum was severed at the distal anal tail attachment, and all of the mesorectums were excised. The rectum was cut at the position below the tumor using the abdominal linear cutting obturator, and then about 5 cm was cut into the abdomen to enter the abdomen, where the sigmoid colon was cut. The proximal pouch was used to tighten the rectum and then enter the abdominal cavity. After surgical suture, pneumoperitoneum was reset to ensure good anastomosis under direct vision. After air was injected, after passing through the anus to confirm that there is no air leakage in the anastomosis, the abdominal cavity is cleaned. The material in the abdominal cavity was placed in front of the sacrum through a drainage tube and flowed out through the right lower abdominal wall or the perineum.

All patients pulled out the drainage tube 1d after surgery, so that the abdominal cavity gradually returned to normal mechanism. If fluid exudation occurs at the post-operative incision, the drainage tube should be opened in time.

### Observation Indicators

According to the response evaluation criteria in solid tumor (14), the surgical efficacy of patients was evaluated, which could be divided into complete response (CR), partial response (PR), stable disease (SD), and progression of disease (PD), and the total response rate = (CR+PR) cases/total cases ×100%. The clinical data of operation time, incision length, intraoperative bleeding volume, and hospitalization time in the two groups were recorded. About 4 mL of peripheral venous blood was collected pre-operatively and on the 3rd day after surgery, and the serum levels of motilin (MTL) and gastrin (GAS) were detected by radioimmunoassay. The relevant test kits were purchased from Shenzhen Jingmei Biological Technology Co., Ltd. The duration of abdominal distension, the time for the abdominal sound to return to normal, the time for the anal exhaust to normal, and the time for normal food intake were recorded after operation. The patients were followed up for 6 months post-operatively, and the complications during follow-up were recorded.

### Statistical Methods

The results of this experiment were statistically analyzed by Statistical Product and Service Solutions (SPSS) 20.0 (SPSS Co., Ltd., Chicago, USA). The count data were expressed by (rate), and chi-square test was used for their comparison between groups. The measurement data were expressed by (mean±SD), and *t*-test was used for their comparison between groups. *P* < 0.05 indicates that the difference is statistically significant.

## Results

### Comparison of Efficacy Between the Two Groups

**Figure 1:**
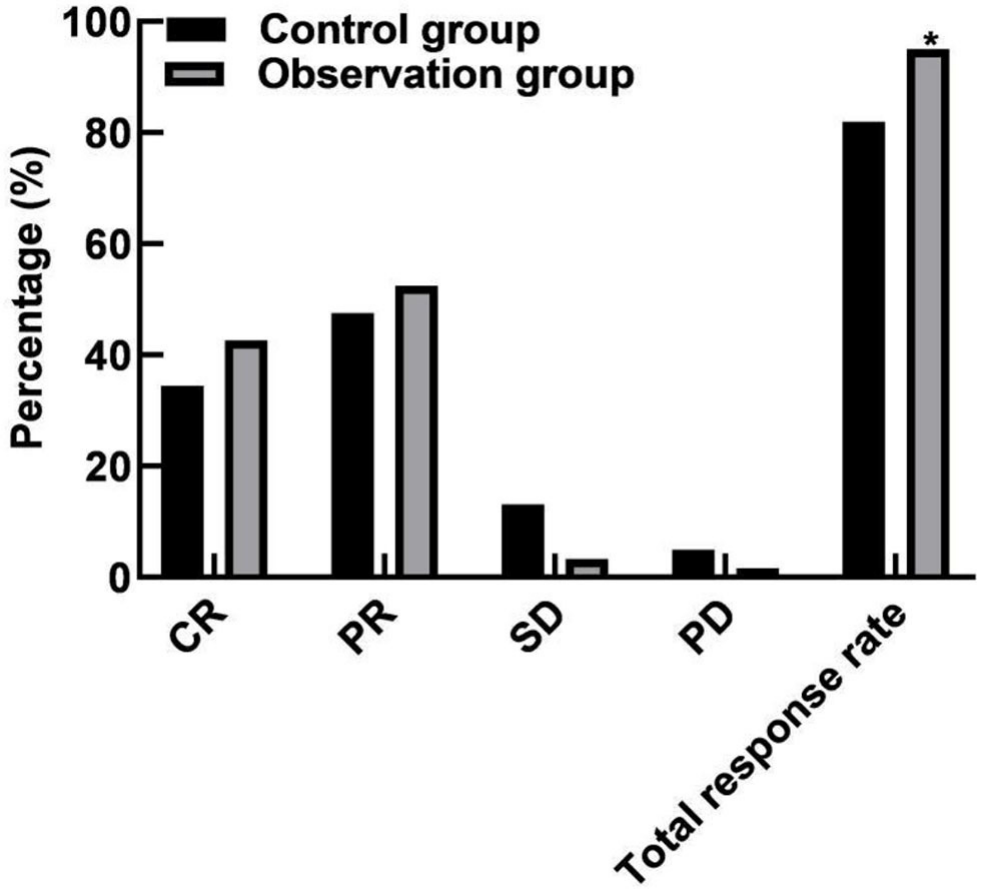
Comparison of efficacy between the two groups. Compared with the control group, *P < 0.05.

In the control group, there were 21 cases of CR, 29 cases of PR, 8 cases of SD, 3 cases of PD, and the total response rate was 81.97% (50/61). In the observation group, there were 26 cases of CR, 32 cases of PR, 2 cases of SD, and 1 case of PD, and the total response rate was 95.08% (58/61). The total response rate of the observation group was higher than that of the control group (*P* < 0.05), as shown in Figure 1.

### Comparison of Clinical Indicators Between the Two Groups

**Figure 2:**
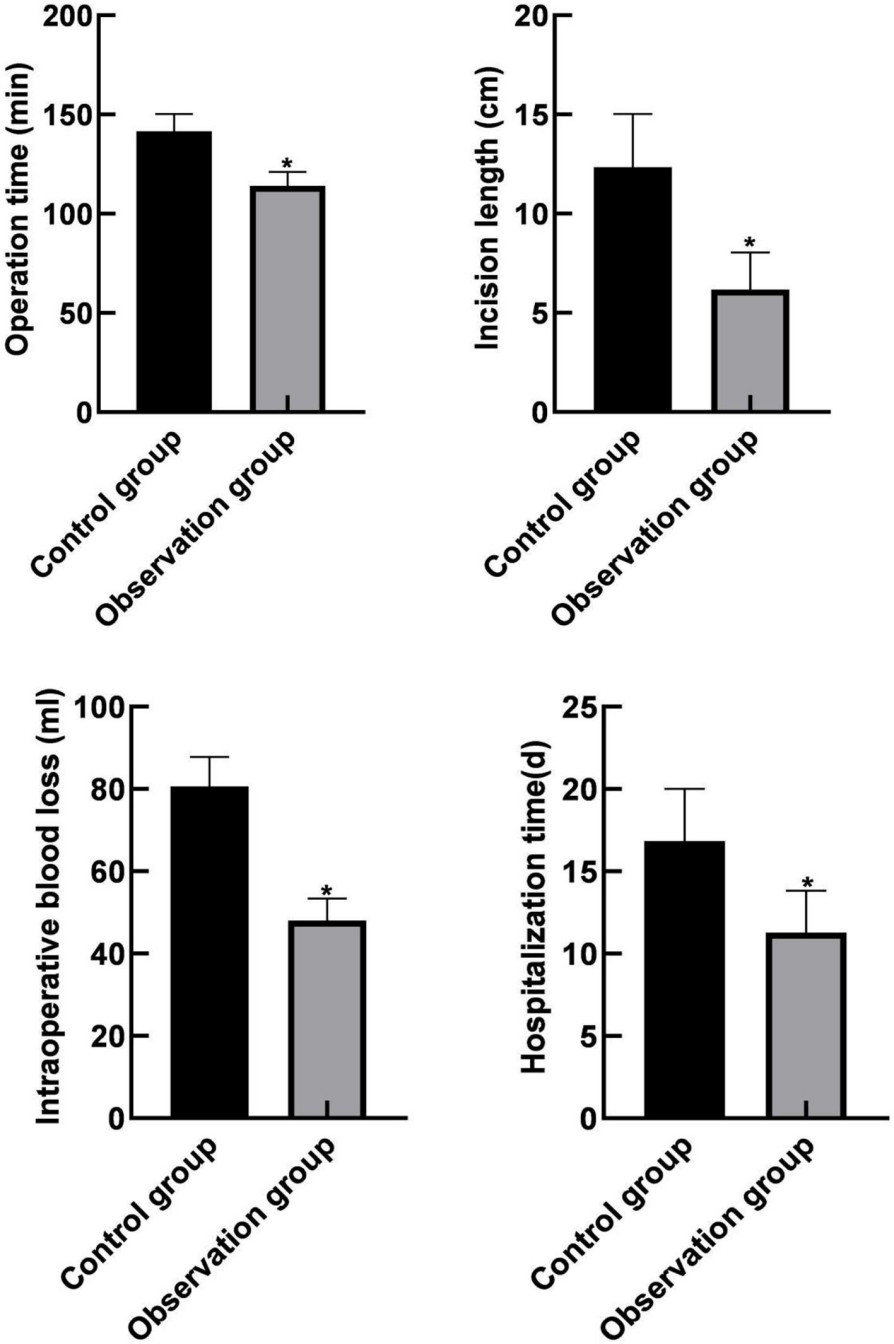
Comparison of clinical indicators between the two groups. Compared with the control group, *P < 0.05.

The operation time, incision length, intraoperative bleeding volume, and hospitalization time in the observation group were lower than those in the control group (*P* < 0.05), as shown in Figure 2.

### Comparison of Gastrointestinal Function Recovery Between the Two Groups

**Figure 3:**
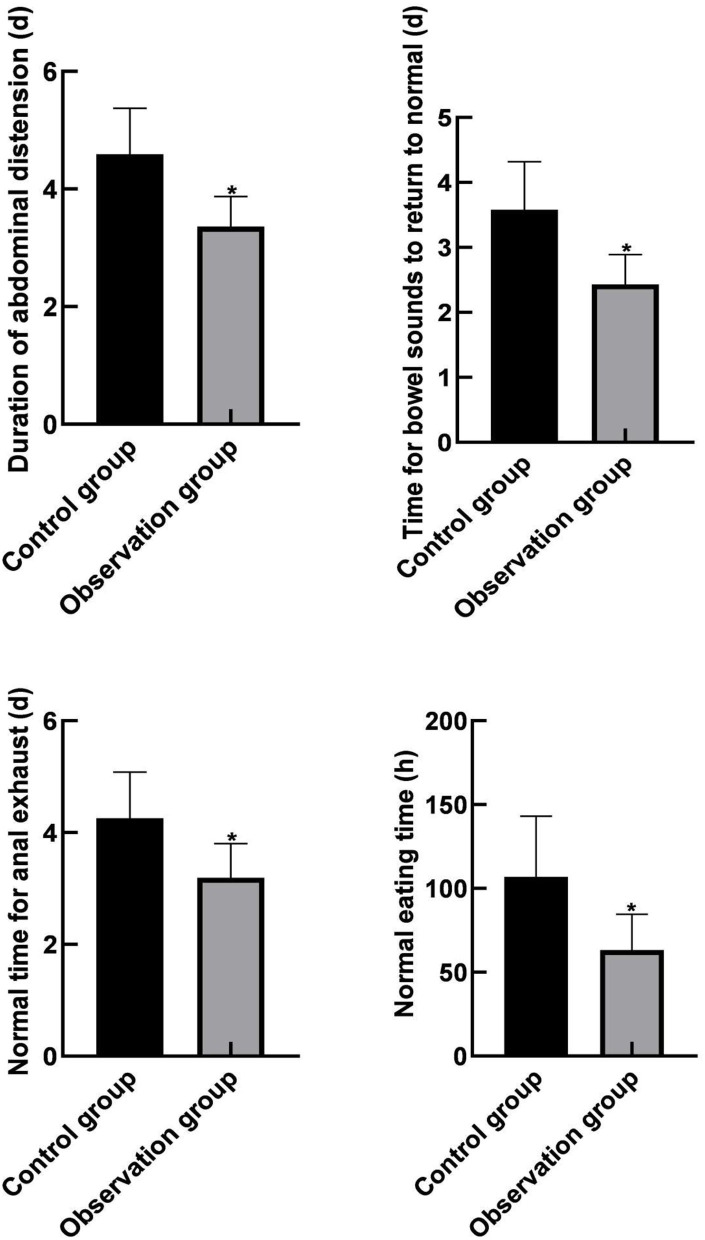
Comparison of gastrointestinal function recovery between the two groups. Compared with the control group, *P < 0.05.

The duration of continuous abdominal distension, the time for bowel sounds to return to normal, the normal time of anal exhaust, and the normal eating time of the observation group were lower than those of the control group (*P* < 0.05), as shown in Figure 3.

### Comparison of Gastrointestinal Function Levels Between the Two Groups

**Figure 4:**
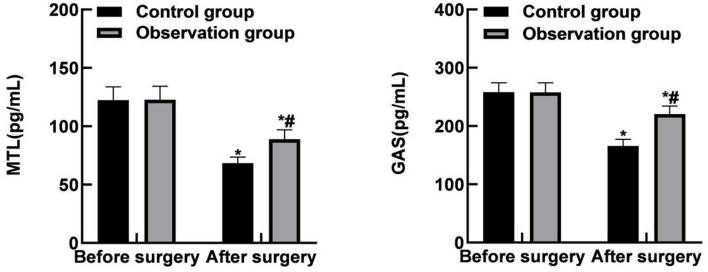
Comparison of gastrointestinal function levels between the two groups. Compared with the same group before surgery, *P < 0.05; Compared with the control group, ^#^*P* < 0.05.

The post-operative MTL and GAS levels of the two groups were lower than those before the operation, and the observation group was lower than the control group (*P* < 0.05), as shown in Figure 4.

### Comparison of Complications Between the Two Groups

**Figure 5:**
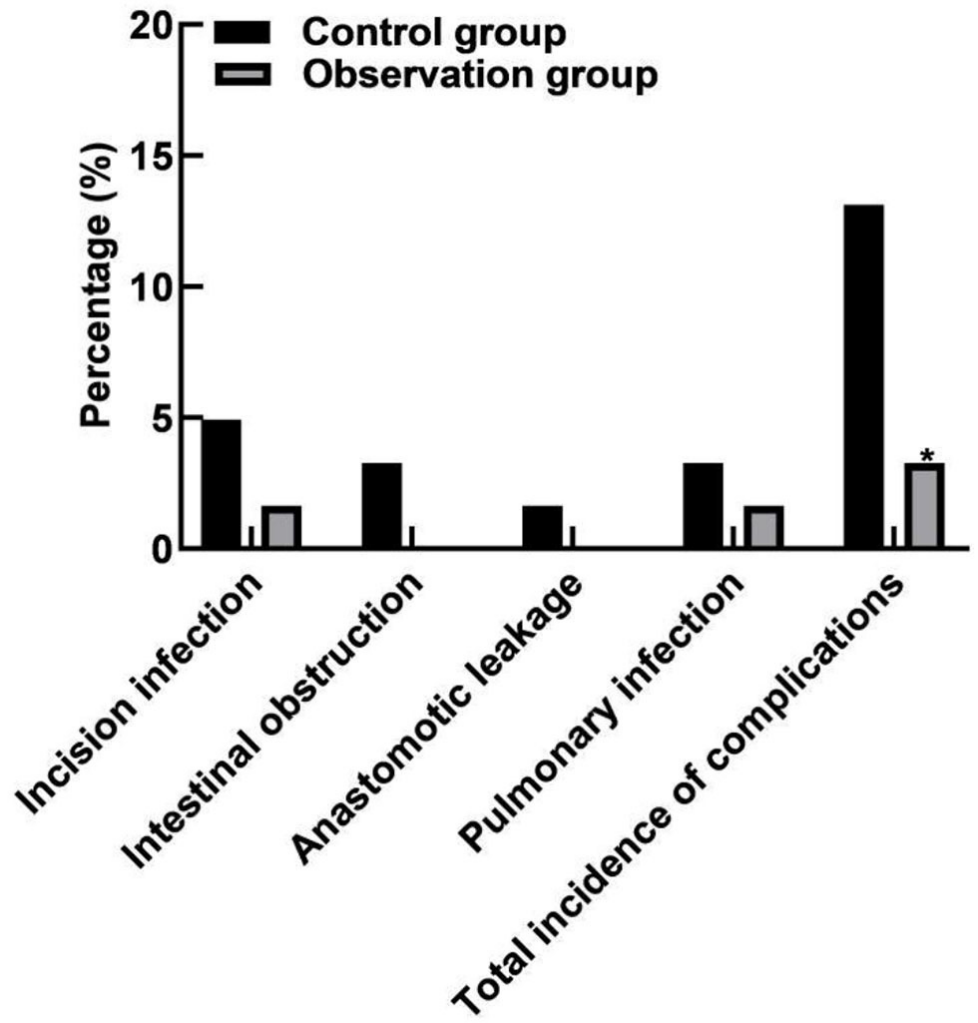
Comparison of complications between the two groups. Compared with the control group, *P < 0.05.

The total incidence of complications in the observation group (3.28%) was lower than that in the control group (13.12%) (*P* < 0.05), as shown in Figure 5.

## Discussions

In recent years, with the improvement of multidisciplinary comprehensive treatment and diagnosis including surgery, radiotherapy, pathological diagnosis, and imaging diagnosis, as well as the development and application of cytotoxic drugs and highly selective molecular targeted drugs, the survival time of patients with colorectal cancer has been significantly prolonged (15–17). Neoadjuvant therapy can reduce the local recurrence rate of some stage II and III rectal cancers and improve the 5-year survival rate of patients. However, as far as the current medical level is concerned, the most effective treatment is still early radical surgery (18–20).

The results of this study showed that the total response rate of the observation group (95.08%) was higher than that of the control group. And the operation time, incision length, intraoperative bleeding volume, and hospitalization time of the observation group were lower than those of the control group. This indicates that compared with the traditional laparotomy, laparoscopic radical resection for colorectal cancer has the advantages of shorter operation time, less trauma, less intraoperative bleeding, shorter hospitalization time, and better curative effect. The reasons were analyzed as follows: Compared with traditional surgery, laparoscopy has the effect of enlarging the visual field, which can make the visual field of laparoscopic radical surgery for colorectal cancer clearer. It is beneficial for laparoscopic multi-angle observation of adjacent tissues, revealing that the anatomical space is not easy to be observed during laparotomy. The trauma is small, and it only needs a few small openings in the abdomen. During the operation, the inflammatory reaction was mild, the amount of bleeding and fluid loss was small, and the internal environment was relatively stable in laparotomy. Post-operative patients had mild pain response, rapid recovery, and significantly reduced exhaust time and total hospitalization time as compared with those after laparotomy (21–23). Moreover, the results of this study showed that the duration of abdominal distension, the time for bowel sounds to return to normal, the time for normal anal exhaust, and the time for normal food intake in the observation group were all lower than those in the control group. After operation, the levels of MTL and GAS in the two groups were lower than those before operation, and the levels in the observation group were lower than those in the control group. These results indicated that laparoscopic radical resection of colorectal cancer could effectively promote the recovery of gastrointestinal function in patients. The analyzed reason is that the recovery speed of gastrointestinal function in patients with colorectal cancer after surgery is related to surgical trauma and stress, while laparoscopic surgery itself has the characteristics of small incision and small trauma, which can avoid the effects of the above factors on the recovery of gastrointestinal function in patients to a certain extent (24–26).

The results of this study showed that the total incidence of complications in the observation group (3.28%) was lower than that in the control group (13.12%). All these have indicated that laparoscopic radical resection for colorectal cancer in the elderly patients has less complications and high safety. The reasons were analyzed as follows: Large incision in laparotomy, poor blood circulation around the incision, and reduced anti-infection ability; meanwhile, the chance of infection was increased due to the long healing time and increased number of dressing changes (27–29). For one case of anastomotic leakage in the control group, it was probably related to too little free anastomosis broken end, excessive traction of anastomosis intestinal wall, local contusion, lax suture caused by improper use of cutter and stapler, excessive tension of anastomosis outlet, or poor blood supply around anastomosis. There were two cases of pulmonary infection in the control group and only 1 case in the observation group, which may be caused by post-operative incision pain in patients afraid of severe cough, resulting in sputum retention in the throat and increasing the chance of pulmonary infection. However, the incision in the laparoscopic group was significantly smaller than that in the open surgery group, so the incision pain in the laparoscopic group was lighter. In order to avoid the occurrence of lung infection, the patients can be instructed to stop smoking and strengthen breathing exercise 6 weeks before surgery, strengthen chest deep breathing, avoid the fixation or binding of limited breathing after surgery, encourage expectoration, sputum can be given drugs to assist expectoration when expectoration is difficult, and sputum suction device can be used when necessary.

Laparoscopic radical resection of colorectal cancer has the same indications as laparotomy, but not all patients are suitable for laparoscopic surgery (30, 31). For example, for patients with long-term heart disease or lung disease, because they cannot perform pneumoperitoneum treatment for a long time, traditional open surgery must be used. For patients with intestinal obstruction or severe abdominal complications after surgery for certain diseases, laparoscopic surgery can easily cause intestinal dilatation, severe congestion, and difficult surgery, resulting in secondary infection during the operation. For patients whose bleeding cannot be controlled, laparoscopic radical surgery is easy to cause surgical anatomy is not smooth, the field of vision is fuzzy, and cannot effectively complete the laparoscopic surgery. Before the start of laparoscopic surgery, if there is a serious adhesion problem in the abdominal cavity, it is easy to cause intestinal damage such as bleeding in the laparoscopic surgery (32, 33). After summing up the experience of the author's department after surgery, it is believed that the specific location of the tumor should be accurately located before the operation to ensure the smooth completion of the operation. During laparoscopic surgery, try to avoid traction on the tumor, and use cotton tape to ligate the mesentery and tumor intestine to prevent the spread of cancer cells. When separating cancer cell tissue, try to handle it in the interstitial space to avoid intestinal injury. When the tumor is removed, it should be cut in a certain size and range in strict accordance with the requirements of the surgery. It should also be ensured that the cancer cells around the lymph nodes and related tissues are completely removed to ensure that the colon cancer can be cured and the cancer recurrence rate is minimized. Post-operative gauze and thin film bags should be provided to complete the protection of the intestinal incision, and to seal the sleeve to ensure no air leakage. The blood plasma drainage tube should also be retained and located not far from the anastomosis so as not to compress the anastomosis. Through observation of the drainage liquid at the anastomosis, we ensured that the drainage tube could smoothly conduct drainage and prevented the occurrence of inflammation. Because laparoscopic surgery loses the tactile feedback of fingers, and the operation space is narrow, the operation area is sometimes located in the deep pelvic cavity, the operator's hands are far away from the target unit in the operation area, and the exposure of the target unit in the operation area is difficult, which makes the colon dissociation and lymph nodes dissection more difficult than traditional surgery. Therefore, it is necessary to make full preparations before operation.

## Conclusions

Laparoscopic radical resection of colorectal cancer in the elderly patients has good effect, short operation time, less trauma, less blood loss during operation, short hospital stay, good recovery of gastrointestinal function, fewer complications, and high safety.

## Data Availability Statement

The original contributions presented in the study are included in the article/supplementary material, further inquiries can be directed to the corresponding author/s.

## Ethics Statement

The studies involving human participants were reviewed and approved by the Ethics Committee of Cangxian Hospital. The patients/participants provided their written informed consent to participate in this study.

## Author Contributions

BL wrote the manuscript and is the instructor of the entire research for comprehensive guidance. BL, CY, and HL collected the clinical datas and do statistical analysis. All authors contributed to the article and approved the submitted version.

## Conflict of Interest

The authors declare that the research was conducted in the absence of any commercial or financial relationships that could be construed as a potential conflict of interest.

## Publisher's Note

All claims expressed in this article are solely those of the authors and do not necessarily represent those of their affiliated organizations, or those of the publisher, the editors and the reviewers. Any product that may be evaluated in this article, or claim that may be made by its manufacturer, is not guaranteed or endorsed by the publisher.
